# Sleep in Isolated, Confined, and Extreme (ICE): A Review on the Different Factors Affecting Human Sleep in ICE

**DOI:** 10.3389/fnins.2020.00851

**Published:** 2020-08-11

**Authors:** Pierpaolo Zivi, Luigi De Gennaro, Fabio Ferlazzo

**Affiliations:** Department of Psychology, Sapienza University of Rome, Rome, Italy

**Keywords:** sleep, ICE, circadian rhythms, stress, polar environments, hypoxia, microgravity, spaceflights

## Abstract

The recently renewed focus on the human exploration of outer space has boosted the interest toward a variety of questions regarding health of astronauts and cosmonauts. Among the others, sleep has traditionally been considered a central issue. To extend the research chances, human sleep alterations have been investigated in several analog environments, called ICEs (Isolated, Confined, and Extreme). ICEs share different features with the spaceflight itself and have been implemented in natural facilities and artificial simulations. The current paper presents a systematic review of research findings on sleep disturbances in ICEs. We looked for evidence from studies run in polar settings (mostly Antarctica) during space missions, Head-Down Bed-Rest protocols, simulations, and in a few ICE-resembling settings such as caves and submarines. Even though research has shown that sleep can be widely affected in ICEs, mostly evidencing general and non-specific changes in REM and SWS sleep, results show a very blurred picture, often with contradictory findings. The variable coexistence of the many factors characterizing the ICE environments (such as isolation and confinement, microgravity, circadian disentrainment, hypoxia, noise levels, and radiations) does not provide a clear indication of what role is played by each factor *per se* or in association one with each other in determining the pattern observed, and how. Most importantly, a number of methodological limitations contribute immensely to the unclear pattern of results reported in the literature. Among them, small sample sizes, small effect sizes, and large variability among experimental conditions, protocols, and measurements make it difficult to draw hints about whether sleep alterations in ICEs do exist due to the specific environmental characteristics, and which of them plays a major role. More systematic and cross-settings research is needed to address the mechanisms underlying the sleep alterations in ICE environments and possibly develop appropriate countermeasures to be used during long-term space missions.

## Introduction

Human factor research has always been a primary topic of interest in the space agencies’ programs. However, the recently renewed focus on the human exploration of outer space has boosted the interest toward a variety of questions regarding safety for astronauts. Traveling to another planet or being involved in a long duration space-mission requires individuals to deal with several exceptional conditions (e.g., microgravity, altered light-dark cycle, danger, autonomy, isolation, and confinement) whose effects on psychophysiological processes are still not well understood. Humans can adjust to these conditions, but in a way that it is still not well-known, making this knowledge strictly necessary for the development of effective countermeasures to support the success of a mission. Consequently, human adaptation in extreme conditions has been investigated in several analog (to space) environments, called ICEs (Isolated, Confined, and Extreme). ICEs share different features with the spaceflight itself and have been implemented in natural facilities and artificial simulations. The advantage of using ICE environments mostly relies upon the reduction in costs and the increase in practicability relative to space missions, and thus the possibility to program long-term experimental projects. However, they vary widely in both the degree and the nature of the challenges they present to individuals. Moreover, it is reasonable to argue that all the challenges can give different outcomes if combined or presented alone. In this picture, deciding which research focuses should be mainly adopted and developing a reliable theoretical framework are of primary importance.

Among others, sleep in space has traditionally been considered a central issue, and great attention is still being paid to it (e.g., Pandi-Perumal and Gonfalone, 2016). Generally, sleep in space has been found to be shorter in duration and worse in quality than on Earth (Evans-Flynn et al., 2016b; Wu et al., 2018), but the causes, the degree, and the functional role of this apparent impairment are still not clear.

Spaceflights and analogs are characterized by the presence of systemic (i.e., having more physical qualities, such as hypoxia, environmental noise, and low physical activity) or processive (i.e., having more psychological features, such as lack of privacy, boredom, fatigue, and interpersonal issues) stressors (Herman and Cullinan, 1997). The effects of stress on sleep have been characterized by neurogenic and somatic responses (Buguet et al., 1998), and often their interaction makes their individual effects quite indistinguishable. Moreover, the environmental circumstances put severe constraints on experimental control: in most studies, the sample sizes are small, and it is often necessary to rely on only subjective measurements.

Unfortunately, the small number of studies in each specific environment, the variety of experimental conditions, and the typically small sample sizes prevent any meta-analytic approach to be adopted. Thus, this review aims at summarizing the findings on human sleep in ICE environments by reducing, as much as possible, the different environments considered to the least common denominators.

This will be done by reviewing the results obtained in the most investigated ICEs (spaceflights, polar environments, HDBR, and Mars analogs) which made use of objective measures of sleep (polysomnography -PSG- and actigraphy). Thereafter, the principal findings of sleep studies investigating the effects of specific factors that individuals in ICEs might encounter will be described. We will consider: circadian disentrainment, as observed in caves, submarines, and forced desynchrony studies; noise, as experienced in Intensive Care Units and laboratory controlled exposures; altered levels of oxygen, such as in high-altitude environments and hypo/hyperbaric chambers; psychosocial stressors.

Finally, the findings reviewed will be discussed, together with the different possible strategies for studying sleep in ICEs, suggesting a possible enlargement of the spectra of relevant experimental conditions. Clarifying the incidence of specific factors rather than investigating the broader effect of the prolonged permanence in ICEs may represent a central issue in this field.

## ICE Environments

Isolated, Confined, and Extremes are very complex environments in which the simultaneous presence of various type of stressors may hide their specific effects on humans’ physiology. The knowledge about the factors present in each, and their relative weight, is of paramount importance for the understanding of the outcomes observed in experimental studies. Hereafter, a brief description of the most investigated ICEs is presented.

### Spaceflight

Human exploration of the outer space has never been a concrete objective of national governments and international space agencies as nowadays. Even though the previous presence of other space stations (such as the Mir, the Skylab, and the Salyut), the construction of the International Space Station (ISS) has definitely permitted the uninterrupted presence of human beings in space allowing for long-term multinational collaborations in orbit (Kanas and Manzey, 2008). The activities performed on this orbiting laboratory are precious for the astronauts training for manned exploration of space. For example, long-term microgravity may affect individuals’ physiological systems, the free-fall experience may affect sensorimotor abilities, and the multiple daily revolutions around the Earth of the ISS may have a strong impact over astronauts’ circadian rhythmicity (Frantzidis et al., 2019). Together with the presence of other stressors such as constant noise, radiations, and prolonged isolation, the astronauts’ health in orbit is constantly threatened. Increasing knowledges about the direction of changes in space will improve our understanding of how organisms cope with these stressors and prepare safe missions to planets, satellites, asteroids, and outer space.

### Polar Environments

Since several decades, Antarctica has been the most productive place were analog studies have been conducted. The presence of dozens of research stations, the uncontaminated and harsh environment, the reproduction of long-term confinement due to the difficulty to reach almost all stations during the winter, and the circannual alteration of seasonality are just some of the features that make it an exceptional context to study for psychophysiological research. A key element in polar environments is the quantity of light to which individuals are exposed during a day. Beyond the 66°33′ parallel (North or South), there is at least one day in which the Sun stay above and one below the horizon for 24 h. Proceeding throughout latitudes, this continuous day/night may spread to six months at 90°.

Moreover, Antarctica is the coldest continent, where temperatures may be lower than −80°C. Furthermore, some stations are located at high elevation, in some cases above 4000 meters above the sea level, making hypoxia another important threatening condition. Besides coping with all these physical factors, crewmembers have to deal with the interpersonal issues that may arise in very small and often heterogeneous groups of individuals (Suedfeld and Weiss, 2000).

The opposite Pole presents similar conditions but is quite more reachable. Indeed, some of the lands above the Arctic Circle are inhabited by civil human communities even though remote research stations are also present in the harshest areas, such as in Nunavut (Canada), Greenland, Svalbard Islands, and in Yakutia (Russia).

### Head-Down Bed Rest (HDBR)

Space agencies have developed HDBR protocols to investigate on Earth the physiological effects of weightlessness. In these protocols, individuals must rest for days, weeks, or months on a tilted-down bed (usually 6°) to reproduce the adaptation of human bodies as in space. HDBR protocols impose reduced physical activity and body fluid shift, possibly inducing adverse biomedical effects such as bone demineralization, cardio-vascular deconditioning, and muscle wasting, typical conditions experienced under exposure to microgravity.

### Mars Analogs

Due to the programmed travel to Mars, several research projects have been implemented by space agencies as real mission simulation on Earth. Among these, the Mars-500 experiment has been one of the most productive.

Mars-500 has been a precious program to study sleep in a controlled condition of prolonged confinement. The experiment consisted of a pilot study (105 days) and a main study (520 days) in which an international crew of 6 men lived and worked inside an isolation facility located at the Institute of Biomedical Problems (IBMP, Moscow). The facility consisted of four hermetically sealed habitat modules, plus one external module simulating the Martian surface. During both the studies, crewmembers participated in several experiments wherein sleep was also investigated.

Up to now, other important simulation studies come from two NASA stations: the Mars Arctic Research Station in the Canadian Arctic and the Mars Desert Research Station in Utah.

### Other ICEs

Several other ICEs exist on Earth, such as caves, submarines, and dry immersions, even though studies conducted in them are still very few. For instance, despite the early interest for circadian investigation in caves and subterranean environments, these contexts have just been recently included among the space analogs (Strapazzon et al., 2014). Together with submarines, caves are an example of exceptional environments wherein a circadian misalignment might appear due to the lack of sunlight exposure. In addition, caves and submarines provide total isolation from the outer world: individuals can’t use any natural time-cue and are forced to interact with few individuals in a restricted environment. For this reason, NASA’s project “NEEMO” aims at investigating underwater environments as space-analogs. In dry immersions, instead, volunteers stay in a sort of bathtub for several days in suspension to simulate the floating conditions of spaceflight.

Widening the spectra of ICEs is really important to make adequate comparisons between conditions. Specific factors must be analyzed alone and together with the others to investigate their peculiar and mixed effect on human health.

## Literature Selection

A literature search for studies investigating sleep in ICE environments was conducted in the Scopus and PubMed databases and in the NASA Technical Report Server using the keywords *sleep* followed by *ICE environment*, *extreme environment*, *spaceflight*, *Antarctic(a)*, *Arctic*, *polar*, *head-down bed-rest*, *HDBR*, *Mars*, and closely related terms. Additional articles were identified from reference lists. Studies that made no use of objective measures (polysomnography and actigraphy) were not included, as well as high-altitude studies in Antarctica (hypoxia has been treated as a separate specific factor).

## ICEs and Sleep

The literature selection process resulted in 13 spaceflight, 18 polar, 6 HDBR, and 5 Mars analog studies. They will be reviewed in this paragraph evidencing common findings and limitations.

### Short-Term Spaceflights

Astronauts’ sleep in space is threatened by many factors, the most prominent being confinement, space radiation, microgravity, noise, artificial light exposure, workload, and shifts in schedules. Gravity is the most studied as it is known, through animal and human research, to have a role in REM sleep: indeed, it has been proposed that the REM onset is associated with the reduced sensitivity to environmental parameters, such as the gravity force (see Gonfalone, 2016 for a review). Interestingly, research on animals has shown that aquatic mammals exhibit strongly reduced or absent REM sleep, whereas semi-aquatic mammals show a reduced REM sleep when in water, and a REM sleep comparable to most of the terrestrial mammals when out of water (Madan and Jha, 2012b). Those results on animals may help to understand the impact on REM sleep of spaceflight conditions, which have been linked to the presence/absence of gravity (Gonfalone and Jha, 2015).

Several subjective reports indicated that sleep duration and quality (Dijk et al., 2001; Kelly et al., 2005; Barger et al., 2014a) are affected during spaceflight.

In an early PSG study, Gundel et al. (1993) described, in a single astronaut in an 8-days space mission, a reduction of both REM latency and REM amount in the second sleep cycle, whereas SWS was redistributed from the first to the second cycle. This study firstly suggested a possible effect on the temporal organization of sleep rather than on the duration of sleep stages, even though it was conducted on just one astronaut.

Differently, Monk et al. (1998) recorded the sleep EEG activity in 4 astronauts who spent 17 days in space during a shuttle mission. The authors employed two 72h measurement blocks during the flight, from the second day in space (early), and from the 12th day (late); also, there were a pre-flight block in the week before the launch, and a post-flight block two weeks after the return. The pre-flight measure was used as the baseline in only one participant (because of the highly stressful week before the launch), whereas the post-flight measure was used as the baseline for the other three astronauts. The first sleep episode in each block was used as habituation. The authors reported no changes in REM sleep, but reduced sleep duration and slow-wave sleep (SWS) during the spaceflight, compared to the baseline. However, the authors did not run any statistical analyses, given the small sample size.

Dijk et al. (2001) reported in five astronauts during the STS-90 and the STS-95 shuttle missions that in-flight sleep was similar to pre-flight sleep for all the PSG parameters but the latency to REM that was shorter in-flight than pre-flight. However, a marked variability was found once sleep episodes were splitted into three segments, with significant effects mostly concerning the three post-flight recordings. Overall, they found that in the first post-flight session REM latency was markedly reduced compared to pre-flight measurements but returned to the in-flight levels in the second and third post-flight sessions. However, after the return to Earth the wake percentage decreased in the first segment, the SWS percentage decreased in the second segment, and the REM percentage increased in the first and the second segments compared to pre-flight. Even if not significantly, wake percentage decreased in the first segment also during in-flight, suggesting that sleep onset was not disturbed. Hence, the alterations observed in-flight consisted of a general decrease in REM latency, that prolonged to the post-flight sessions, a decrease in SWS, and an increase in wake during the third segment of sleep, compared to pre- and post-flight and to pre-flight only, respectively. Finally, sleep period time was found to be reduced during spaceflight compared to pre- and post- sessions. It must be noted that the very low statistical power in this study make it likely that at least some of the described effects are due to chance only.

Indeed, Chen et al. (2018) did not find any alteration on sleep-wake activity in astronauts, though sleep quality before and during the flight was lower than that after the space flight. They found a longer but less efficient sleep after the spaceflight than in the pre- and in the in-flight periods, and more efficient sleep and shorter sleep latency during the in-flight period compared to pre- and post-measurements.

Summarizing, the most relevant and surprising finding during and after short-term spaceflights was that the overall structure and temporal organization of sleep were not markedly altered (Table 1), besides some small changes. Overall, PSG sleep parameters during early flight, late flight, and post-flight recordings did not show any difference apart from a drastic decrease of REM latency in the first post-flight sleep. As reported elsewhere (Manzey and Lorenz, 1998), adaptation effects rather than stable systematic changes might occur more prominently in astronauts, especially in short-term exposure to microgravity, requiring more investigations.

**TABLE 1:** Overview of studies investigating sleep through objective measures during space-flight missions.

References	Participants	Conditions	Methods	Main findings
Barger et al., 2014a	Sixty-four astronauts on 80 space shuttle missions (26 flights, 1063 in-flight days) and 21 astronauts on 13 ISS missions (3248 in-flight days), with ground-based data from all astronauts (4014 days).	Space-shuttle missions and International Space Station	Wrist activity and daily logs. Ground-based data for 2 weeks scheduled about 3 months before launch, 11 days before launch until launch day, and for 7 days upon return to Earth.	Crewmembers obtained significantly more sleep per night after the mission compared to during and pre-flight periods in both space-shuttle and ISS missions.
Chen et al., 2018	Three crew members (two males and one female, aged 33–49 years).	15-days space-flight	actigraphy, questionnaires, heart rate. For activity, measurements were obtained in three test sessions for each crew member from −63d to −54d for preflight, from 3d to 14d for flight and from +0d to +8d for postflight.	Longer sleep duration on Earth, especially postflight compared to during and pre-flight. Higher sleep efficiency in-flight. Sleep quality was higher after than before and during the flight.
Dijk et al., 2001	Five astronauts (1 female, 37 to 46 years)	Two shuttle missions (16 and 10 days)	PSG, actigraphy, core body temperature, urinary melatonin and cortisol, subjective sleep quality, and neurobehavioral assessment. PSG recording sessions: Three pre-flight (approximately 3, 2, and 1 month before scheduled launch); four sessions in-flight, two early (3–6 days after launch) and two late flight (12–15 days after launch); three post-flight sessions (second, fourth, and fifth night after landing). Double-blind placebo-controlled melatonin administration.	More wakefulness, less SWS during the final third of sleep episodes, diminished subjective sleep quality in space. The amplitude of the body temperature rhythm was reduced and the circadian rhythm of urinary cortisol misaligned relative to the imposed non-24-h sleep-wake schedule. Neurobehavioral performance decrements were observed. During postflight, REM sleep was markedly increased. Melatonin administration did not improve sleep.
Evans-Flynn et al., 2016b	21 astronauts (15 males)	International Space Station, 6-month space mission	Actigraphy and photometry data were collected over 3,248 days of long-duration spaceflight on the ISS and 11 days prior to launch (n = 231 days).	In orbit, sleep duration, sleep quality was shorter, and use of sleep-promoting medications higher during misaligned sleep episodes than during aligned episodes. Estimated endogenous circadian temperature minimum occurred outside sleep episodes on 13% of sleep episodes during preflight and on 19% of sleep episodes during spaceflight.
Frost et al., 1976	Three astronauts	Three 28-, 59-, and 84-day space missions	PSG	Stage 3 sleep increased during the flight and decreased in the postflight period; stage 4 was consistently decreased postflight, although this stage was variable during the flight; stage REM (rapid eye movement) was elevated, and REM latency decreased in the late postflight period (after day 3 post-recovery); and the number of awakenings during sleep either showed no change or decreased during the flight.
Gundel et al., 1993	1 astronaut	8 days space-flight (6 days in orbit). For comparison the same parameters were measured during a 5-days baseline period preceding the space mission.	PSG (nights 3–7), questionnaires, body temperature	In space REM latency was shorter, there was less REM sleep in the second non-REM/REM cycle, and slow-wave sleep was redistributed from the first to the second cycle. Circadian rhythms of body temperature and alertness were delayed.
Gundel et al., 1997	Four astronauts aged 39-53.	MIR station	PSG, body temperature. Two to seven PSG recordings during the first 30 days in-flight. Baseline measures were taken up to 6 days and at least 4 weeks before the launch.	In Space, sleep was shorter and more disturbed than on Earth. The latency to the first REM episode was shorter SWS was redistributed from the first to the second sleep cycle. The circadian phase of body temperature was found to be delayed by about 2 h in space compared with baseline.
Moldofsky et al., 2000	8 male cosmonauts/astronauts (mean age 45.5 years)	MIR station	Sleep and endocrine functions were investigated during baseline data conditions preflight. Five subjects who served on MIR for 4 to 6 mon. were studied during mid-flight and late-flight at about days 81, 82, 143, 144, then 1wk.post flight. Three to six mon. later, 3 subjects participated in post-flight follow-up.	Within flight, time in slow wave (deep) sleep declined. Increased awake time, increased movement arousals to stage 1 sleep, and decreased % stage 1 sleep during late flight vs. preflight. The first week postflight vs. preflight was associated to increased sleep period time, increased awake time, reduced sleep efficiency, and reduced % stage 2 sleep.
Monk et al., 1998	Four astronauts (age 38-47, mean 42.5)	17-days space-flight mission	PSG, core body temperature, urinary melatonin and cortisol, actigraphy, sleep diaries, mood and alertness VAS, and questionnaires. Four 72h measurement blocks: preflight (starting 7 days before launch), early flight (starting 2 days after launch), late flight (starting 12 days after launch), and postflight (starting 18 days after landing).	In-flight sleep showed a decreased amount of sleep obtained, and all four astronauts showed a decrease in delta sleep.
Petit et al., 2019	Five astronauts (mean age 53 ± 1.6)	International Space Station, 6-month space mission	Seventy-min wake EEG recorded between 2 and 10 hours after awakening. A visuo-motor task was administred.	Global wake theta oscillations increased on ISS.
Stickgold and Hobson, 1999	Five astronauts	NASA 4 and Russian MIR 23	PSG, subjects recorded an average of 26 nights of sleep during the preflight period, 24 nights inflight, and 14 nights postflight during the postflight recovery period. All told, 317 nights of sleep data were recorded, of which 120 were collected inflight, between the 24th and 171st day in orbit. Sixty of the 120 nights were recorded more than three months into the flight.	For all five subjects, REM sleep was severely diminished during flight. In contrast, postflight REM was similar to preflight. There was also decrease in total sleep time and sleep efficiency inflight.
Stoilova and Jordanova, 2005	A total of 27 recordings of cosmonauts’ sleep	MIR station	PSG	Compared to normative values, a very slight decrease of SWS and dramatic reduction of REM sleep in the pre-flight session was observed. During the space-flight, SWS remained low and REM markedly increased.
Stoilova et al., 2000	Five astronauts	MIR station. Astronauts spent 9, 10, 43, and 241 days in-flight. One astronauts was recorded only before flight.	PSG	In Space, sleep latency shortened relative to baseline. SWS latency was lengthened after several months of space flight.

Adaptation effects, indeed, have also been reported in subjective measures: debriefed astronauts reported that the duration of their sleep was shorter on the first and the last day of the mission (Santy et al., 1988). Other subjective reports indicated a trough also after three months compared to the first observation (Monk et al., 2001), even if other evidence reported that sleep quality worsened during the first part compared to the last part of the mission (Stuster, 2010). However, general impairments were not found (Whitmire et al., 2013).

It is not known if the reduced amount of sleep required by astronauts during spaceflight is a temporary effect of spaceflight stressors or a characteristic of the exceptional individuals involved (Evans-Flynn et al., 2016b). Microgravity *per se*, for instance, may affect sleep measures without impairing the ability of sleep to be restful: an HDBR study found that sleep quality was reduced, but the subjective feeling of refreshment was not (Boschert et al., 2019).

Results from sleep studies conducted during long-term missions or in microgravity analogs (such as HDBR) might be useful to disentangle the acute effects of microgravity from the adaptation/long-term effects of isolation and confinement.

### Long-Term Space Missions

The psychophysiological literature aimed at investigating alterations due to the permanence in ICEs has broadly showed that short and long-term effects may coexist. Indeed, two different kind of adaptation processes can be outlined: at the outset, an acute adjustment to the environmental challenges followed by a progressive acclimatization of these first effects and, presumably, by the cumulative development of long-term psychological and physiological consequences. When possible, considering both the processes is essential for the understanding of all the possible outcomes exerted by the permanence in ICEs. Simultaneously, such adjustments might be investigated both pre- and post-permanence, to further disentangle specific changes space-related (such as microgravity) to unspecific adaptation effects.

For instance, due to the different aims that move human beings in space, spaceflights can be of different durations, making possible the distinction between short- and long-term effects on sleep in space, and the relative possible pre- and post- alterations.

Six studies during long-term spaceflights used PSG. Firstly, Frost et al. (1976) observed, during the Skylab mission, an increase/decrease in stage 3 sleep during/after the spaceflight period and a decreased stage 4 post-flight with some variability during the spaceflight. REM sleep increased, and REM latency decreased after the mission. However, the authors suggested that none of the observed changes could be expected to adversely affect performance capability.

Subsequent studies have been mostly conducted on the Russian space station MIR. Gundel et al. (1997) found no significant variations in TST and in sleep efficiency, but a shorter REM latency in the first and increased SWS in the second sleep cycle during in-flight compared to pre-flight baseline on 4 astronauts. However, differences in the time-points when the nights were recorded among the astronauts did not allow to observe possible adaptation effects.

With a different approach, Stoilova et al. (2000) compared short- and long-term astronauts in prolonged space missions, they found a longer SWS latency compared to the baseline and the short-term missions. Subsequently, Stoilova and Jordanova (2005) described a marked REM sleep and SWS decrease in the pre-flight period. During in-flight REM sleep remained low, while SWS increased (also compared to normative values). Both studies, however, remained essentially descriptive without any statistical comparison. Differently, Moldofsky et al. (2000) studied eight astronauts in the MIR space station and observed a reduction of SWS during spaceflight compared to the pre-flight, while Stickgold and Hobson (1999) found a 50% reduction of REM sleep in-flight, together with the decrease of total sleep time and sleep efficiency.

One recent study conducted on the ISS investigated sleep events during wake (Petit et al., 2019). EEG was not recorded during sleep, but, interestingly, they found that the astronauts involved in a docking simulation task presented increased global theta EEG oscillations while in space than on Earth, and that this increment was associated with slower reaction times. It is worth to note that this relationship was found despite the comparable amount of time asleep the night before and time awake before the recordings (even if it was not objectively measured). The investigation of circadian- and homeostatic-related changes in sleep pressure (i.e., subjective and objective measures of sleepiness) during wakefulness appears to have paramount importance for the safety of astronauts’ activities and the development of effective countermeasures.

Actigraphy studies also provided measures of circadian and adaptation changes. The incidence of misaligned sleep has been investigated by Evans-Flynn et al. (2016a) aboard the ISS on 21 astronauts between 2006 and 2011 (missions 14 to 26). They reported that compared to the pre-flight baseline, there were more misaligned sleep episodes relative to endogenous circadian temperature (the authors defined a misaligned sleep episode as the minimum core temperature falling out of the sleep episode) during the spaceflight. Moreover, the authors observed that sleep duration was shorter, and quality was lower during the misaligned than during the aligned sleep episodes. On the other hand, Barger et al. (2014a) compared 64 astronauts who participated in a shuttle mission to 21 astronauts who worked on the ISS. Both groups of astronauts were found to sleep significantly more after their return to Earth than during the pre-flight baseline (up to 3 months before the launch) and the in-flight period.

As for the short-term space studies, also the long-term ones revealed mixed pictures. The most representative findings obtained (Table 1) regard changes in REM and SWS sleep, even if the direction of these effects remains not clear, and the shortening of REM latency during and after the flight, eventually representing a response to adaptation, additive to the circadian adjustment.

Primary analogs on Earth where the additive effects of long-term isolation and circadian adjustment can be investigated are the polar environments. The lack of microgravity (and noise and radiations as well) in these contexts allows for a better understanding of specific and unspecific changes in sleep behavior due to the permanence in ICEs.

### Head-Down Bed Rest Protocols

Head-Down Bed Rest is today the gold standard for the investigation on ground of physiological changes due to microgravity.

Five PSG studies investigated sleep changes due to simulated microgravity conditions, though they differ for the duration and the degree of head-down position. In one of them, 11 participants rested one night in the 12° head-down condition (Boschert et al., 2019). The authors observed a significant increase in light sleep and a significant decrease in SWS and REM sleep in HDBR compared to the horizontal control condition. In more prolonged and less tilted protocols, Komada et al. (2006) found in 7 participants, who stayed 6° tilted down for 7 days, a significant decrease in stage 4 sleep in the former half of sleep and an increase in the number of arousals compared to the pre-ambulatory recordings. In a 17-days study, without daylight exposure, 8 subjects showed a marked increase of sleep latency and percentage of night awakenings in the early phase of bedrest compared to baseline (Monk et al., 1997). However, the authors did not find any significant changes in sleep architecture. Instead, Samel and Gander (1995) studied the effects of 11 days of 6° degrees HDBR and the effects of a phase shift and a bright light illumination treatment on sleep. Their results showed that total sleep time and sleep efficiency were lower than pre-shift during the days of phase shift. Moreover, stage 2 sleep significantly decreased (by 20%), and SWS increased during the shift days and after the resynchronization to the new shift. Moreover, the HDBR group slept less and less efficiently than an ambulatory control group and presented a trend to more stage 1 and less stage 4 sleep. Accordingly, Frantzidis et al. (2020) found a degradation in stage 2 sleep in a 6° and 60 days-protocol. Total sleep time was not found to change in an actigraphy recording of 45-days of 6° head-down bed rest (Liang et al., 2014).

Summarizing, also in the case of HDBR studies, results are controversial, mostly because of the difference in protocols, their duration, and the severity of the tilt (Table 2). It is plausible to argue that more severe and acute exposure to head-down sleep (i.e., simulated microgravity) might exert a pattern of effects that can disappear or change when habituation occurs or when the restricted environment becomes uncomfortable.

**TABLE 2:** Overview of Head-Down Bed-Rest studies investigating sleep through objective measures.

References	Participants	Conditions	Methods	Main findings
Boschert et al., 2019	Eleven male subjects (mean age 30 +/− 10.2 years)	21h 12°tilted down bed rest	Each subject participated in two 3-day (2 nights) campaigns, one week apart. During the second night of each campaign, subjects slept −12° tilted down or horizontally. Besides PSG, assessment included transcranial Doppler ultrasound (TCD), near infrared spectroscopy (NIRS), MRI measurements and a cognitive test battery.	Significant increase in light sleep and decrease in N3, REM sleep and sleep quality in head down compared to horizontal.
Frantzidis et al., 2020	Twenty-three male individuals aged between 23 and 45 years old (mean: 29 ± 6 years).	60-days 6° tilted down bed rest	PSG recorded 14 days before experiment onset and during the 21st day of the HDT period were analyzed. One group of subjects underwent a Reactive Sledge Jump training during the bedrest phase.	Impairments in global network characteristics and their feature connectivity during N2 sleep.
Komada et al., 2006	Seven male subjects (mean age 26 ± 4.5)	3 days (2 nights) 6° tilted down bed rest	Two within subject sessions (head down and horizontal bed rest). PSG was recorded on the third night of each session (after two ambulatory days). PSG, Stanford Sleepiness Scale, Alpha Attenuation Test, Event-related Potential (ERP), cognitive tasks.	Slight decrease in N4 in the former half of sleep for head down bed rest. Number of arousals increases.
Liang et al., 2014	Eight healthy men (mean age 26.2 +/− 4.1 years)	45-day 6° tilted down bed rest.	Actigraphy, ECG. Nine ambulatory days pre- and 14 post-bedrest.	Increased daytime sleep during HDBR. Total sleep time was similar to pre- and post- measures. Changes in heart rate and in high and low frequencies of heart rate variability were observed.
Monk et al., 1997	Eight male volunteers aged 25–51 years old (mean: 42.3 +/− 8.0 years). All but one of them had had prior experience in bedrest studies.	17 days 6° tilted down bed rest. External daylight was eliminated.	Three 72-hour measurement blocks: ambulatory pre-bedrest, early bedrest (days 5-7), and late bedrest (days 15-17). Two weeks pre- and post- bed rest were also part of the study. PSG was recorded in each of the three nights of each block. Body temperature and subjective measures of sleep were also collected.	Augmented sleep latency in the early phase and percentage of time spent awake during the night. Reduced subjective sleep quality. Temperature rhythms showed reduced amplitude and later phases resulting from the bedrest conditions.
Samel and Gander, 1995	Four healthy subjects, aged 35-41 years. An age-matched control group was also part of the study.	11 days 6° tilted down bed rest. On days 7 and 8, the time awake was extended by 6 h each to result in a total phase delay of 12 h. Subjects were treated by bright light (>3500 lux) for 5 h on those two shift days and the consecutive day, according to a blind cross-over design. In the control condition, the light exposure was centered on the time of the predicted temperature maximum when it was expected to have no phase shifting ability. Under the experimental condition, the light exposure was timed to the end of the daily minimum in body temperature which was predicted from the preceding minimum for each subject.	PSG, body temperature, heart rate, urinary melatonin and electrolytes rhythms, questionnaires	Sleep was shorter and less efficient in HDBR than in ambulatory control group. Decrement in stage-2 sleep and increase in stage-4 sleep during the shift compared to pre-shift.

### Polar Environments

Besides hypoxia in high-altitude Antarctic stations, the alteration of the normal circannual seasonality and the long-term confinement, together with the interpersonal issues related to isolation, are among the major challenges individuals who spend prolonged periods in polar environments face. Unfortunately, the lack of a systematic investigation of seasonal sleep changes through objective measures, in natural non-confined environments, does not permit a clear understanding of the specific role of confinement, and even if a specific role does exist.

In two early polysomnographic (PSG) studies with similar designs investigating an Antarctic crew for 9 and 8 months, respectively, opposite results were found. Paterson (1975), in a study carried out at the Halley station with 10 participants and monthly sessions, found a significant reduction of Slow Wave Sleep (SWS) in winter (August) compared to the first month (April). This decrease lasted until the end of the study, except for the September session. Also, stage-2 sleep, summer Total Sleep Time, and REM sleep were reported to increase, albeit without any supporting analysis. Comparing their results to a previous study with similar findings at the high-altitude South Pole station (Natani et al., 1970), Paterson suggested the incidence of altered light conditions as a major cause for SWS decrement. However, no significant changes in sleep efficiency were highlighted in a subsequent study (Buguet et al., 1987) at the Dumont D’Urville station in 8 (of the 28) crewmembers with usual good sleeping habits; indeed, the results showed an opposite trend: stage 3-4 sleep increased, while stage 1-2 sleep decreased. Buguet et al. (1987) argued that the crewmembers were physically active outside during the winter (whereas Paterson (1975) reported that the physical exercise was greater during the summer) and that acclimation to cold might have played a role. However, the two studies did not include any baseline measurements. The only one-year study (Bhattacharyya et al., 2008) recorded 3 consecutive nights per month for a total of 13 time-points (1 pre-departure) with six male crewmembers at the Indian Maitri station. Compared to the pre-departure baseline, total sleep time (TST), sleep efficiency (SE), and stages 3-4 decreased, whereas wake after sleep onset (WASO), and stage 1-2 increased in Antarctica. These effects appeared almost at all months, except for the November session, when the effects on stage 1-2 and 3-4 were opposed compared to the general trend (i.e., stages 1-2 decreased and stages 3-4 increased compared to the baseline) and recovered toward the end of the expedition. Further seasonal effects were observed in the winter months when the changes of the PSG measures were found to be more consistent, and REM sleep was also found to increase compared to the baseline. Differently, sleep latency exhibits a specific lengthening in January (i.e., summer, but also the first month in Antarctica) and in April.

Seasonal changes in sleep onset and sleep duration have also been reported through actigraphy.

In a large study conducted at the German Neumayer station, on the overwintering crews from 2008 to 2014 for a total of 63 participants (Steinach et al., 2016), both the permanence in confinement and the circannual photoperiod effect showed different effects on sleep-wake activity, depending on gender. Indeed, while changes in sleep efficiency fitted a linear and a quadratic trend, time in bed for all the crewmembers and women alone, sleep onset for all the crewmembers and men alone, and sleep offset for all the crewmembers and also for men and women separately, varied according to seasonal changes, fitting a quadratic trend with maxima in mid-winter. Differently, sleep time significantly decreased linearly throughout the year for all participants, while the number of arousals per night increased for all the crewmembers and women only, suggesting a cumulative effect of the permanence in Antarctica. Sleep duration was found to decrease significantly during midwinter on 13 crewmembers at the Argentinian Belgrano station (Folgueira et al., 2019) compared to other seasonal time-points. However, even in this case, the summertime point was the last month of the Antarctic period, and possible effects other than photoperiod might be present. Instead, total time slept, together with sleep efficiency and latency, did not change in 17 winter-over crewmembers at the Chinese Zhonshang station (Chen et al., 2016). The authors reported a delay of sleep onset, offset, a mid-sleep time in Antarctica compared to a pre-departure measurement, with a peak in midwinter for sleep onset and before and in mid-winter in the other time-points compared to the baseline and to the end of winter (Chen et al., 2016). Delay in sleep timing, and an increase in sleep fragmentation, were also found in winter by Francis et al. (2008). Winter delay for bed-, wake-, sleep start-, and sleep end-time were also observed by Kawasaki et al. (2018). In their study, two Antarctic stations, Halley and Concordia, were investigated, aiming at highlighting possible differences between the two altitudes. The inter-daily stability of the circadian rhythm (the stability over days) declined after two months without direct sunlight, and the 10-hours period with the maximum activity was delayed. The authors reported no relevant differences between the two stations in the circadian and sleep indices. Differently, Collet et al. (2015) found that at sea level (Dumont D’Urville station) individual sleep-time was longer, sleep-efficiency was higher, and wake after sleep onset was shorter than at Concordia, confirming the role of altitude in determining the pattern of results observed in most of the Antarctic studies (Pattyn et al., 2018). With regard to seasonality, their results point to a general worsening of the sleep quality during summer compared to winter, with higher fragmentation and night energy expenditure. The detrimental effect of summer was investigated and confirmed in a recent PSG study (Pattyn et al., 2017). In their study, after 3 weeks of acclimation in Antarctica, 9 subjects were compared to a control group of 12 good sleepers. They found a comparable Total Sleep Time, while sleep onset latency, sleep efficiency, SWS, REM latency, and light sleep were significantly lower, whereas REM sleep, wake after sleep onset, and the latency between the first REM episode and the first SWS episode were higher. Instead, an actigraphy study revealed no effects in summer (Weymouth and Steel, 2013). However, their study was conducted with 14 students on a 12-day field trip in Antarctica, where the expedition members spent 1 night at the Scott Base, 7 nights in a camp several kilometers away, and 3 nights at the Scott Base again. Thus, the design, the nature, and the short duration of the trip, as well as the sample, might have a role in the negative finding.

At the opposite Pole, PSG has been used by only two studies conducted during winter with the primary aim to assess the effects of cold. Buguet et al. (1976) found increased wakefulness, decreased SWS and REM, while Angus et al. (1979) found a 50% decrement in REM sleep in the first night in the Arctic and a 25% decrement in the subsequent nights as compared to the baseline sleep. This large REM reduction further worsened during the coldest nights.

Beyond some confirmations of Antarctic findings, these two studies suggest a specific effect of REM suppression by cold, which resembles the robust phenomenon in animal studies of REM deprivation by exposing animals to cold temperature (Amici et al., 1998, 2008). Thermal stress, due to the alteration of normal sympathetic/parasympathetic activity, exerts a homeostatic change in NREM sleep, with more light and fragmented episodes, and, consequently, an adaptive suppression of REM sleep (Szymusiak, 2018). However, given the technological development of polar stations and facilities, the impact of cold has now been dramatically reduced, if not eliminated.

Together with the Antarctic studies, where light interventions have been revealed effective in reducing total sleep time, advancing sleep start and wake up time (Corbett et al., 2012), reducing sleep onset and sleep latency (Mottram et al., 2011), and augmenting sleep efficiency (Francis et al., 2008), the effectiveness of countermeasures has also been studied in the Arctic. The Arctic studies were implemented in Alert (Paul et al., 2015a,b,c). In a first study (2015a), the authors compared two groups of individuals who spent at least one month *in loco*, one group in winter (January), and one group in summer (June). Results showed that participants in the winter group slept significantly more than those in the summer group. In another study (2015b), two summer groups (in 2012 and 2014) were compared, and melatonin was tested as a countermeasure. First, the authors showed that participants in the second trial slept more than their counterparts in the first trial. The authors reported that schedules and daily-light exposure changed between the two groups, with more conservative station rules during the second campaign, especially regarding light exposure restriction during the night. However, the melatonin treatment did not exert any macroscopic change in the objective sleep parameters. Differently, the last study was conducted during winter and involved individualized light-intervention based on melatonin circadian-rhythm (2015c). In the post-treatment measurement, sleep efficiency and percentage of sleep were higher, and wake after sleep onset and number of sleep episodes were lower than the pre-treatment session, with no effects on sleep quantity.

Summarizing, studies in polar environments mostly suggest a general worsening of sleep quality as indexed by decreases of SWS (i.e., the sleep stage more strictly associated to homeostatic need), TST and sleep efficiency, and a concomitant increase of sleep fragmentation (i.e., WASO) and lighter sleep stages (i.e., stages 1 and 2). Seasonal effects have also been shown.

Until now, it is not clear how a prolonged permanence in a confined polar environment affects sleep. Again, the findings reviewed (Table 3) might be attributable to seasonal changes, to the confinement itself, or to a mixture of the two. Unfortunately, very few studies aimed at investigating their differential role, for instance by methodologically disentangling linear from seasonal effects. Hence, more studies aimed at comparing sleep changes at different stations, at different altitudes and latitudes, are strongly needed. Furthermore, considering other laboratory-controlled studies aimed at investigating specific effects of circadian disentrainment on sleep stages, as well as investigations in natural but not confined polar or quasi-polar environments will be highly informative on how and how much sleep changes are dependent upon these specific factors.

**TABLE 3:** Overview of studies investigating sleep through objective measures in polar environments.

References	Participants	Conditions	Methods	Main findings
Angus et al., 1979	6 males (19–30 years)	Arctic (Churchill, Canada)	PSG. Five baseline, 16 experimental (cold exposure), and 4 recovery nights. Vigilance tests were also administred.	REM sleep decreased in the Arctic compared to baseline. Deprivation increases in the first night and in most cold nights. Reaction times increased, especially in the coldest nights, and did not recover.
Bhattacharyya et al., 2008	6 healthy male crewmembers (mean age 35.7 +/− 2.32 years)	Antarctica (Maitri)	PSG, 1 baseline record + one per month in Antarctica. Total sleep recordings: 234 nights (6 subjects × 3 nights each × 13 months)	TST (in all months), sleep efficiency and stages 3-4 decreased (in winter), wake after sleep onset, stage 1-2 and REM increased (winter months), sleep latency increased immediately after exposure
Buguet et al., 1976	2 caucasian males (age 19 and 22)	Arctic	PSG, heart rate, and body temperature. Two baseline nights, 10 cold exposure nights and 2 recovery nights.	Increased wakefulness, decreased SWS and REM
Buguet et al., 1987	8 participants (of the 28 crew members) with usual good sleeping habits (mean age 29.6 ± 2.6 years)	Antarctica (Durmont D’Urville)	17-21 nights recordings for each subject. The subjects were registered only in Antarctica from April to December. Recording sessions lasted 2-3 consecutive nights.	Did not reveal any variation of sleep efficiency with time. All sleep variables were within normal range. Stage 3-4 sleep increased, while stage 1-2 sleep decreased.
Chen et al., 2016	17 healthy males (age range 23–54 years; mean age 36.2 ± 9.5 years). The data from 11 participants were analyzed	Antarctica (Zongshan Station)	Wrist actigraphy and sleep logs, at least 14 consecutive days. Four time-point: before departure and during residence at Zhongshan Station for 1 year (before winter, mid-winter, and end of winter)	Delayed sleep onset in mid-winter compared to pre-expedition; delayed sleep offset and midtime sleep in mid-winter and before winter compared to pre-expedition. No significant differences in total sleep time, sleep efficiency, or sleep latency were seen.
Collet et al., 2015	26 subjects (5 females, mean age 36 ± 26.5 years).	Antarctica (Dumont D’Urville and Concordia)	Actimetric recordings, 2 time-point: summer and winter	At sea level (Dumont D’Urville station) individual sleep-time was longer, sleep-efficiency was higher, and wake after sleep onset was shorter than at high-altitude (Concordia Station). Higher number of sleep fragmentation episodes and night energy expenditure in summer compared to winter with no interaction between seasonality and location.
Corbett et al., 2012	12 (1 female), median age 30 years (range 21–50).	Antarctica	Actigraphy, sleep diaries, subjective VAS, cognitive performance, and urinary melatonin rhythm. Six weeks at either side of midwinter (2 weeks control period − 4 weeks light intervention − 2 weeks control).	Advanced circadian phase, sleep start- and wake up- time during light intervention compared to control periods. No differences for the other sleep parameters.
Folgueira et al., 2019	13 male army personnel (mean age 34 ± 1 years), one excluded	Antarctica (Belgrano II)	Actigraphy, questionnaires, and psychomotor vigilance tasks. Five time-points (March, May, July, September, November), 7 days recording each	Mean sleep duration significantly decreased during the polar night (July).
Francis et al., 2008	9 men and 1 woman (mean age 33 ± 7 years). Data from 10 subjects (Antarctic) and from 3 to 8 subjects (baseline) were suitable for analysis	Antarctica (Halley)	Wrist activity and light monitors Actiwatch intervention. Sleep diaries daily. 152 ± 17 days data from each subject, 1 control period − 6 intervention periods − 1 control period. A baseline study with no intervention (seven men and one woman, aged 30 ± 7 years) was also implemented.	A clear delay in sleep timing, particularly the end of sleep, was evident in winter. Sleep fragmentation increased. All the sleep periods during light treatment show decrements in one or more attributes of sleep, specifically timing, fragmentation and efficiency.
Kawasaki et al., 2018	25 healthy crew members (mean age: 34 ± 11). Analysis included data from 20 subjects.	Antarctica (Halley and Concordia Station)	Activity watches. Seven months for analysis (one month before, three months during May-July, and three months after the time without direct sunlight August-October. A total of 3017 days (n = 20) was used for analysis.	Winter delay for bed-, wake-, sleep start-, and sleep end- time. No relevant differences between the two stations in the circadian and sleep indices. The inter-daily stability of the circadian rhythm declined.
Mottram et al., 2011	15 (5 females), mean age 32.5	Antarctica	Actigraphy (n = 12), sleep diaries (n = 15), general health, and urinary melatonin rhythm. One control period, 6 alternating blue/white light intervention periods, 1 control period	Reduced sleep onset and latency during blue light intervention compared to the white one. No differences for sleep efficiency. Longest sleep during control condition, followed by blue light and then white light.
Paterson, 1975	10 (mean age 25 years)	Antarctica (Halley Station)	PSG monthly sessions	SWS reduction (in winter, August) compared to the first month (April). It would have continued into the significant increases in amounts of stage-2 sleep matched the reduction in SWS indicating that sleep had lightened. Summer increases in TST and absolute amounts REM.
Pattyn et al., 2017	21 healthy male subjects, free of medication, between 27 and 57 yr (mean age 35 years) and good sleep control group consisted of 12 healthy, medication-free men, aged between 20 and 43 years (mean age: 29 years). Only data from 9 subjects were suitable for sleep scoring.	Antarctica (Princess Elisabeth)	PSG, 18-h profile (saliva sampling every 2 h) of cortisol and melatonin. Mood, sleepiness, and subjective sleep quality were assessed, and the psychomotor vigilance task was administered. The study was conducted during summer.	High sleep fragmentation, a major decrease in SWS and an increase in REM. The ultradian rhythmicity of sleep was altered, with SWS occurring mainly at the end of the night and stage R sleep at the beginning. Cortisol secretion profiles were normal; melatonin secretion showed a severe phase delay. No mood alterations, but an impaired vigilance performance.
Paul et al., 2015a	Two trials (January and June). The January trial included 14 subjects, 9 men and 5 women, with an age range from 21 to 56 years (mean age 36.9 ± 2.9 years). The June trial included 12 different subjects, 8 men and 4 women, with an age range from 22 to 47 years (mean age 31.2 ± 2.8 years)	Arctic (CFS Alert)	Actigraphs, salivary melatonin	Sleep duration was significantly shorter in summer. There were no significant differences in the objective measures of sleep under melatonin treatment
Paul et al., 2015b	Baseline experiment was conducted at CFS Alert from June 8th to 17th, 2012. Trial 1 included 12 subjects, eight men and four women age range from 22 to 47 years (31.2 ± 2.8 years. Trial 2 occurred from May 24th–June 15th, 2014 at CFS Alert, 15 subjects (11 males and four females, age range of 19 to 47 years, 28.3 ± 8.3 years).	Arctic (CFS Alert)	Actigraph, Sleep log, salivary melatonin	The avoidance of nocturnal light is likely to improve sleep during the Arctic summer
Paul et al., 2015c	13 subjects (8 males and 5 females, age range of 22–42 years, mean agef31.2 ± 6.3 years), but one male subject was excluded. Individuals with a melatonin rhythm that was in disaccord with their sleep schedule were given individualized daily light treatment interventions based on their pre-treatment salivary melatonin profile. Data collected from January 10th to February 2nd, 2014.	Arctic (CFS Alert)	Questionnaires, actigraphs data, salivary melatonin	The light treatment prescribed to seven of the twelve subjects was effective in improving sleep quality both subjectively, based on questionnaire results, and objectively, based on the actigraphic data.
Steinach et al., 2016	n = 54 (17 females, median age 33)	Antarctica (Neumayer Station II and III)	Actigraphy, twice a month. Total of measurements: 860.	The decline in local sunshine radiation led to a 48 minutes longer time in bed, lower sleep efficiency, a delay in sleep onset, a delay in sleep offset, and less daily energy expenditure for all participants in reaction to the Antarctic winter’s darkness phase. Gender differences for some sleep parameters were found.
Weymouth and Steel, 2013	14 (8 females) post-graduate students, mean age 36.9 (14.4) years	Antarctica (Scott Base and surroundings)	Actigraphy. Four nights before departure, 1 night at Scott Base, 7 nights at campsite, 3 nights at Scott Base	No significant effects on sleep measures.

### The Mars Analogs

Several attempts have been made to investigate the biomedical and psychological effects of a simulated mission to Mars, especially focusing on the prolonged isolation and confinement, on the scheduling of activities and operations, and on the interpersonal issues that astronauts may encounter during their travel to outer space.

During the Mars-105 experiment, Barger et al. (2014b) observed that both crewmembers and mission controllers obtained inadequate sleep on the day of the nightshift and that sleep deprivation affected cognitive performance, highlighting the importance of adequate scheduling of activities to safeguard the level of performance from sleepiness and stress-like responses during operation of paramount importance. Following this line, Gemignani et al. (2014) found in Mars-105 that higher cortisol levels in the crewmembers were correlated with the reduction of REM latency, the decrease of sleep duration, the increase of arousals, and the reduction of delta power in stage 3 sleep. Instead, in the 520-days experiment, Zavalko et al. (2013) found an increase in sleep and SWS latency when the confinement started. Sleep latency persisted high even after the confinement ended, while SWS latency recovered to the baseline levels before the end of the confined period. In terms of stage percentages, the authors found an increase in stage 2 and a decrease in SWS after the confinement compared to the last part of the isolation period. Crewmembers slept more toward the end of the mission and exhibited less sleep activity in the third quarter of the mission compared to the others (Basner et al., 2013).

Apart from the Mars-500 experiment, sleep has been studied in other simulation of Mars expeditions. For instance, the Hawaii – Space Exploration Analog and Simulation was conducted for 365 days in a geodesic dome in the Mauna Loa volcano’s harsh environment in Hawaii. In a pilot study (Matsangas et al., 2017) evaluating 4 out of 6 crewmembers, high interindividual variability was observed. Whereas no differences in sleep bedtime patterns compared to pre-mission baselines were found at a general level, but the authors reported that the participant with less sleep obtained was more satisfied with his sleep quality, while the opposite was true for the participants who slept more. Moreover, the authors investigated if individual light exposure and physical activity may contribute to the variability observed. For instance, the participant who rested and woke up earlier than the other participants was more exposed to white light during the day, while the participant who slept less but more efficiently was exposed to blue light during the night and was the less physically active.

The astronauts supposed to spend time on Mars have also to deal with the Martian Sol, the daily timekeeper consisted of 24 h and about 39 min per day. With this aim, in Gríofa et al.’s (2011) study, 7 subjects spent 110 days during the Arctic summer at the FMARS station on Devon Island, Canada. After 51 days of confinement, the crew underwent the Martian Sol implementation with no other terrestrial timekeeper. They collected subjective and objective (cardiopulmonary) sleep data. Only non-significant minor variations in subjective sleep indices and Total Sleep Time were found throughout the mission, except for an increase of alertness in the early phase of the Martian Sol compared to the pre-Sol baseline measure. Cardiopulmonary data showed only a tendency to decreased sleep stability during the Sol.

In a short-term study conducted at the Mars Desert Research Station, Utah, during the “AustroMars” mission, Groemer et al. (2010) investigated sleep in 4 crewmembers during two weeks of Mars simulation activities. Since they found no significant changes in sleepiness (as assessed by the Karolinska Sleepiness Scale, KSS) or in vigilance (as assessed by Psychomotor Vigilance Task, PVT), findings that authors attributed to the high motivation showed by subjects during the study.

In conclusion, the few studies conducted (Table 4) until now do not significantly contribute to the already little knowledge produced in ICE environments. However, promising lines of research look possible.

**TABLE 4:** Overview of studies investigating sleep in Mars analogs.

References	Participants	Conditions	Methods	Main findings
Barger et al., 2014b	Six male crewmember participants (four Russian, one French, and one German), aged 25–40 (32.8 +/− 5.6 years). Nineteen Russian mission controllers (nine females), composed of eight engineers and 11 medical personnel, also participated.	Mars-105. Crewmembers were required to perform mission operations daily throughout the 105-day study composed of daily work operations and an extended c duration 24-h work shift every sixth day. Mission controllers were scheduled to work periodic 24-h shifts in support of the mission. Crewmembers are encouraged to nap for about 2 hrs in the afternoon prior 24h shift night; to use caffeine during operations; were provided supplemental light exposure.	Wrist activity, sleep logs, cognitive performance, salivary melatonin	Both crewmembers and mission controllers obtained inadequate sleep during shifts. Decrements in cognitive performance were observed. Melatonin levels during the extended 24-hrs work shifts remained elevated, although countermeasures were taken.
Basner et al., 2013	6 healthy men (3 Russians, 2 Europeans and 1 Chinese), aged 27 to 38 years.	Mars-500	Wrist actigraphy, Psychomotor Vigilance Task	Crew exhibited less activity across the mission and increased sleep and rest times. Light exposure decreased during the mission. The majority of crewmembers also experienced one or more disturbances of sleep quality, vigilance deficits, or altered sleep–wake periodicity and timing.
Gemignani et al., 2014	Six healthy right-handed male volunteers (mean age 33 ± 6 years)	Mars-105, ambient temperature was maintained constant at 24 °C, and an environmental noise in line with that of the International Space Station (60–70 dB) was constantly present	PSG, urinary cortisol, cognitive assessment. 5 time-points: baseline, performed pre-simulation; three equally spaced timepoints during the 105 days simulation; recovery, performed post-simulation.	Higher cortisol levels were associated to: decrease of sleep duration, increase of arousals, and shortening of REM latency; reduction of delta power and enhancement of sigma and beta in NREM N3; and left lateralization of delta activity (NREM and REM) and right lateralization of beta activity (NREM).
Gríofa et al., 2011	Seven crewmembers (3 women), aged 23 to 38 years	110 d during the summer at FMARS on Devon Island, Canada. A 24h and 35min central “Martian” clock was designated for 37 days during the mission. All other station timekeeping objects were cleared for the duration of the Sol.	Sleep diaries, cardiopulmonary, cognitive performance	Sleep diary data indicate improvement in alertness with the onset of the sol. Cardiopulmonary data suggest sleep instability, though trends were not statistically significant.
Groemer et al., 2010	Six carefully selected “analog astronauts”	Two weeks AustroMars mission	Pupillographic sleepiness test, salivary assay data (DHEA, Cortisol, P17-OH, Fasting Insulin and MB2S), cognitive performance, Fatigue Monitoring System.	No significant changes in sleepiness and vigilance
Matsangas et al., 2017	Four of the crewmembers volunteered to participate in the pilot sleep study (24 to 33 years of age, two males), scientists and engineers. They had been living and working for six months in the HI-SEAS habitat at the beginning of the study	12-month Hawaii Space Exploration Analog and Simulation mission	Actigraphy, activity logs	Individual differences in sleep-related behaviors, physical activity and exposure to light between the crewmembers
Zavalko et al., 2013	6 healthy men (3 Russians, 2 Europeans and 1 Chinese), aged 27 to 38 years.	Mars-500	PSG recordings (each consisted of two consequent nights) were performed 10 weeks before, 4 times during and 2 weeks after the isolation. During the experiment 63 suitable polysomnographies were recorded.	During the isolation sleep efficiency and delta latency decreased, while sleep latency increased mainly one month and half before the end of the isolation. Frequency of the nights with low sleep efficiency significantly increased before simulation of Mars landing and the end of the confinement. Two weeks after landing simulation the amount of the nights with low sleep efficiency significantly decreased.

## Analogs of the Analogs

Taken together, the results described above do not appear to highlight a consistent pattern of changes in sleep in ICE environments. The small sample sizes and the large differences existing in the experimental designs and environmental factors indeed represent one of the most prominent causes. The severe methodological limitations existing in this research field posit the question of how it could be possible to advance the knowledge on how sleep adjusts in these exceptional environments, where adequate control conditions are not often possible to implement. One possibility to overcome this weakness is to gather information from other conditions on Earth that might be taken as a reference for the sleep effects identified in ICEs. Even though some of them cannot inherently be qualifiable as ICE environments, environments such as submarines, caves, prisons, military contexts, offshore platforms, intensive care units, hypoxic environments, circadian shift studies, etc., may represent useful comparisons in the progressive construction of a theoretical framework for investigating sleep under a prolonged systemic and/or processive stressful condition.

One main objective of the comparison could be the replication of results obtained when subtracting one of two factors in the condition studies. The next section will describe results reported in various fields, mostly regarding the effects of circadian disruptions and other stressors on sleep.

### Circadian Analogs

Given that crews living and working at polar stations must deal with a circannual change of the normal photoperiodicity and that on the ISS astronauts experience many sunset and sunrises each day, it is not surprising that circadian alterations may arise in individual physiology, as it has been highlighted in several chronobiological studies (Arendt and Middleton, 2018; Premkumar et al., 2013; Harris et al., 2011; Dijk et al., 2001). Indeed, the circadian rhythm is extremely sensitive to light exposure, and with the massive use of artificial lights and digital devices the physiological sleep/wake pattern related only to sunlight exposure has been blurred (Wright et al., 2013). For instance, in indoor workplaces, the light intensity is approximately 300–500 lux, the direct sunlight approximately 100000 lux and 1–100 lux when the sun is below the horizon (Arendt, 2012): low light intensity, even in controlled light-dark cycles, has found to delay sleep onset (Middleton et al., 2002). Though the nature of the possible misalignment seems to be very different in the several environments investigated (e.g., polar stations and space missions), considering other literature can help disentangle the circadian from the effects of other stressful factors on sleep.

For instance, in 19 marines involved in a 70-day mission aboard the French Strategic Submarine with Ballistic Nuclear missiles, Trousselard et al. (2015) did not find any significant difference in any parameters recorded between two PSG sessions (days 21 and 51 of the mission) nor in night-time cortisol levels. The authors concluded that the imposed scheduled activity and the control of light and temperature aboard were important factors for preventing any sleep impairment. In any case, because no control groups or baseline sessions were recorded, no significant conclusion can be drawn. In addition, together with but differently from Gemignani et al. (2014), a correlation between cortisol and sleep has been described: here, cortisol levels were positively correlated to REM sleep after 51 days of the mission. Several other studies conducted and reported by the U.S. Naval Postgraduate School (Miller et al., 2011) provide evidence that submariners sleep subjectively less while underway and that more compressed daily activity schedules resulted in a less amount of sleep, as measured by actigraphy. The findings that work schedules in submarines have an impact on sleep duration have been confirmed elsewhere (Duplessis et al., 2007): social and work times can represent strong *zeitgeibers* for sleep/wake rhythm, even in the absence of sunlight (Yoneyama et al., 1999). In the same report (Miller et al., 2011), other results have been summarized. For instance, on surface ships, mariners have been found to sleep less if working on topside compared to the ones that slept below decks, thus not seeing sunlight, and that rank position might have a role (senior personnel slept less than junior personnel). A recent paper reviewed subterranean studies (Mogilever et al., 2018), not only presenting evidence of the well-known circadian stabilization at more than 24h of several physiological measures (free-running), but also of changes in sleep architecture. Indeed, SWS and REM sleep were found to increase at the expense of light sleep: this happened when individuals were found to sleep desynchronized and to follow a bi-circadian rhythm (34/14h of wake/sleep) (Chouvet et al., 1974). In this case, given the homeostatic role of SWS, the increase in SWS sleep could be more related to the longer time spent awake rather than to the isolation itself.

Circadian misalignments *per se* have been mostly investigated through laboratory-controlled forced desynchrony studies. In two time-isolation studies (10 and 12 days, respectively), Monk et al. found that a progressive 2h phase delay (Monk et al., 2004) led to a significant linear decrease/increase of time spent asleep/WASO during and after the phase delay sessions and a decrement/increment in sleep latency during/after the isolation relative to the baseline, whereas a progressive 0.5h phase advance (Monk et al., 2006) led to a significant decrease/increase of time spent asleep and sleep latency in the last sleep episodes relative to the baseline. However, in both studies no differences were found in REM and SWS percentages. One concern can be advanced over the suited methodology, and over the number of EEG derivations used: in Monk et al.’s as in other space-mission studies (for technical purposes), EEG was only registered at C3 or C4, as reported by the authors. Indeed, it has been shown that SWS has a topographical distribution with dissociable sleep pressure-dependent and -independent components (Bersagliere et al., 2018), and with relevant changes might not be detected. For instance, Lazar et al. (2015) described how sleep-dependent and circadian-related changes of SWS can be topographically distinguished through a forced-desynchrony protocol: frontal areas seem to be most related to sleep- while circadian-related effects might be prominently found in central and posterior areas. In addition to SWS, a circadian role in REM sleep has also been documented (Czeisler et al., 1980).

### Noise

A good example of an environment constituted by a multiplicity of factors that may affect sleep and psychological well-being is the Intensive Care Unit (ICU). High environmental noise, exposure to only artificial lights, low physical activity, social confinement, and lack of privacy are the most common ICU patients’ complaints. However, given the nature of the context, ICU studies have been conducted on clinical populations, in which the pathological condition represents a confounding variable. Keeping that in mind, patients in ICU showed decreased SWS and REM sleep and increased awakenings (Boyko et al., 2012). The only study investigating the effects on healthy participants (nurses and doctors) found similar results when participants were sleeping in the ICU compared to when they were sleeping at home or in an unknown environment (Reinke et al., 2019).

Noise has been considered one of the most influential detrimental factors for sleep in ICU. Given that it is also present during space-missions, attention must be paid to it. Changes in sleep structure have also been found due to laboratory-controlled environmental noise both in animals (Rabat et al., 2004) and humans (Basner and Samel, 2005; Griefahn et al., 2006). Basner and Samel (2005), investigated the effects of nocturnal aircraft noise on sleep. They polygraphically recorded 13 consecutive nights in 128 subjects. Subjects were exposed to aircraft noise events in 9 nights, preceded by an adaptation and a baseline night, and followed by two recovery nights. Noise exposure was related to sleep fragmentation, as stage 1 sleep and number of awakenings increased, whereas SWS decreased. Dose-response relationship between Sound Pressure Level and number of events per night were observed. Similarly, Griefahn et al. (2006) investigated the influence of different road, rail, and aircraft noise during three weeks in 24 participants. Compared to a quietly sleeping control group, participants exposed to noise obtained less sleep and spent more time awake after sleep onset. In addition, SWS and REM sleep decreased even though this effect was not found in the first sleep cycle. Strongest effects were found according to noise levels, while the type of noise exacerbated different changes in sleep structure. The impact of other noise sources has been largely documented: the effects of traffic noise on sleep have also been reported to reduce SWS (Myllyntausta et al., 2020) and influencing the hormonal stress reaction mechanisms (Thiesse et al., 2020). Similar effects were shown during night exposure to wind turbines (Ageborg Morsing et al., 2018). In general, transportation noises have been positively associated with the probability of stage transition to wake or Stage 1 (Basner and McGuire, 2018).

### Hypoxia

A prominent physical stressor that has been widely investigated in relation to sleep is the availability of oxygen. Most of the studies have been aimed at investigating sleep apnea phenomena, but literature also exists on the investigation of natural and laboratory-controlled oxygen deprivation. In many circumstances (such as some Antarctic stations), the oxygen level might fall below normal values for human well-being. Environmental levels of oxygen are related to barometric pressure, which mainly depends upon altitude. The higher the altitude, the lower the barometric pressure, the lower the partial pressure of oxygen (PO2): this condition is named hypobaric hypoxia. People who work and live in high-altitude environments experience several physiological, neurological, and psychological effects (Wilson et al., 2009). Organism response to hypoxia acts as a compensatory mechanism. When PaO2 (arterial pressure of oxygen) falls below ∼60 mmHg, the carotid body, through chemoreceptors, starts a signaling cascade that involves the nucleus of the tractus solitarii, the ventrolateral medulla and the paraventricular nucleus of the hypothalamus. These structures are central regulating autonomic activity: the increase of the alveolar ventilation (HVR, hypoxic ventilator response) and the sympatho-adrenal activity (peripheral epinephrine levels and cardiovascular activity) are the first responses that allow the maintaining of the oxygen supply. Hyperventilation leads to periodic breathing during sleep, which is the hypothesized mechanisms through which sleep (mostly light sleep) is affected under hypoxic exposure (Ainslie et al., 2013). Cerebral blood flow increases due to vasodilation induced by catecholaminergic and non-catecholaminergic processes: central nervous system and cognition can be affected, as it is broadly described in the high-risk literature (Virués-Ortega et al., 2004; Taylor et al., 2015; McMorris et al., 2017). Several studies also suggest that acute hypoxia can determine an increase in cortisol levels (Heyes et al., 1982; Simeoni et al., 2011). Differently, chronic exposure to hypoxia may result in an attenuation of these symptoms, in a process known as acclimation or acclimatization. For instance, Strewe et al. (2017) found that chronic exposure to hypoxia (21 days) did not affect individual stress response. Moreover, because a high percentage of the studies conducted in Antarctica have been conducted in high-altitude internal stations (e.g., Concordia station), the effects of hypoxia on sleep might have been confounded or strengthened the effects due to the photoperiod alteration and to confinement (Pattyn et al., 2018). In these studies, the hypoxic environment was found to increase sleep fragmentation compared to sea-level (Collet et al., 2015). SWS was also found to be reduced (Natani et al., 1970; Shurley, 1974), together with REM sleep, and sleep onset latency (SOL) increased (Natani et al., 1970), but in these studies there was no comparison with a sea-level station. A recent study (Mairesse et al., 2019) reported that sleep parameters investigated through PSG were found to be stable throughout a one year-campaign at the Concordia station, with increased averaged SOL compared to literature standards. Accordingly, besides respiratory variables and positive effects of exercise, sleep has been found not to change in a long-term permanence at the Concordia Station, even if REM sleep has been found to increase in a compared short-term hypoxic exposure after 10 days in a normobaric chamber (Tellez et al., 2016). The increase in REM sleep has been suggested to be a regulatory mechanism helping the organism to maintain optimum levels of CO2 through the modulation of breathing response, as has been observed in the switch between NREM and REM sleep in normal sleep (Madan and Jha, 2012a).

Shorter TST was found in a real high-altitude environment and in a simulated environment compared to a control condition (Heinzer et al., 2016). Accordingly, in a simulated normobaric hypoxic experiment (Pramsohler et al., 2019) it was reported that the more severe the hypoxia, the lower the TST, together with reductions in REM sleep. Comparisons between bed rest and hypoxic conditions showed that SWS decreased while light sleep increased due to hypoxia (Rojc et al., 2014; Morrison et al., 2017). Coherently, studies in high-altitude treks revealed similar patterns (Nussbaumer-Ochsner et al., 2011).

Research on hyperbaric environments also shown similar results. For instance, Stoilova (1992) found that prolonged exposure to increased pressure in 40 aquanauts decreased SWS and REM sleep percentage, paralleling other results observed in space and analogs. Similar findings were provided by a precedent study (Rostain et al., 1988). Apart from changes in sleep stages, increased WASO and SOL, and decreased TST and SE were commonly observed in these studies (Seo et al., 2001; Nagashima et al., 2002a). However, similar results were also reported for a non-pressurized control group in a subsequent study (Nagashima et al., 2002b): the authors concluded that results were mostly due to a reaction to the psychological stress of confinement.

Lighter sleep and more fragmentation seem to be the principal findings for the hypoxic studies. However, as it has already been written, hypoxic effects in Antarctica might be confounded by other challenging conditions. Comparisons between high-altitude and sea-level investigations, together with simulated hypoxic environments, might be the right way to solve the puzzle. Moreover, problems in identifying the specific effects of hypoxia or, conversely, cut them off to isolate photoperiodicity and isolation, may also arise when considering acute and chronic exposure of oxygen deprivation and, thus, the counteractive impact of acclimation.

### Psychosocial Stressors

With regards to purely psychological variables, few results have been produced. Comprehensively, psychosocial stress has also been showed to reduce SE, SWS, and REM, and to increase awakenings, even if the findings are not definitive given the large number of different stress conditions studied and the small number of studies conducted on sleep (for a review, Kim and Dimsdale, 2007). In their review, Kim and Dimsdale (2007) divided everyday stress from laboratory-induced stress. They observed that works studying the effects of everyday emotional stress lead to a reduction of SWS. They reported that a reduction of SWS was also found in laboratory-induced stress, both for mild stressor, such as using an indwelling venous catheter (Prinz et al., 2001) or the “first night effect” (Curcio et al., 2004), and emotional stress (Levin et al., 2002), whereas conflicting evidence for REM has been reported. However, high-risk environments can frequently be characterized by a lack of privacy and interpersonal conflicts, and a link between sleep disturbances and lack of privacy has been suggested (Jaksic et al., 2019). An early study conducted in prison found a reduction in TST, SL, and percentage of stage 2, and an increase in awakenings in prisoners compared to normal controls, but no changes in REM and in SWS (Karacan et al., 1974). Studies on rats on the effects of social defeat stress reported that the conflict itself caused an increase in SWS and a suppression of REM sleep in the first hours after the conflict (Kamphuis et al., 2015).

Despite the low number of studies and the variety of conditions investigated in the field, the results obtained have shown that sleep structure can also be altered by moderate psychological stressors. Astronauts and individuals who live and work in confined environments, in very close prolonged contact with other few individuals reported that factors regarding interpersonal relations and communication with the outside are often challenging, representing one of the most prominent stressors in ICE. According to current knowledge, linking those variables to sleep problems looks premature, and future research must be able to keep track, as possible, of personal and interpersonal stressful events during the permanence in ICE, to understand eventual variations in sleep indices better.

## Discussion

This review aimed at drawing a current state of our knowledge on human sleep in ICE environments by comparing findings from studies run in different settings. Results show a very blurred and contrasting picture, with very few apparently consistent effects, and essentially no clear indication of which of the many factors characterizing the ICE environments play a role *per se* or in association with each other.

Regarding spaceflights, the few existing studies reported that during short-term space missions, astronauts show a decreased SWS, an increased REM, and reduced sleep efficiency (e.g., Monk et al., 1998; Dijk et al., 2001). Albeit these alterations mimic those reported in Antarctica, contradictory findings have also been reported (e.g., Chen et al., 2018). Specific, albeit small, changes in REM and SWS also occur during long-term space missions, even if the direction of these effects remains unclear.

Some hints about the factors determining the sleep alteration in ICEs might come from the studies performed in polar environments (mostly, Antarctica), that share the isolation and confinement condition with space-flight missions. The reviewed studies suggest, but not univocally, a general worsening of sleep quality as indexed by the decreased slow wave sleep (SWS) (i.e., the sleep stage more strictly associated with homeostatic need) and sleep efficiency, and the concomitant increase of sleep fragmentation (i.e., WASO) and lighter sleep stages (i.e., stages 1 and 2) (see Pattyn et al., 2018, for a recent review). However, it is not clear how a prolonged permanence in a confined polar environment determines these changes and if these changes are a specific or aspecific (e.g., acting as a stressor) consequence of polar confinement. Indeed, they might be due to seasonal changes, the confinement itself, a mixture of the two, or any other feature of the polar environment. Unfortunately, very few studies aimed at investigating their differential role, for instance by methodologically disentangling linear from seasonal effects.

Bed-rest studies, simulating microgravity’s effects on physiological and psychological processes, are hard to interpret because of differences in protocols and duration. However, even in this condition, studies exist that report decreased SWS and increased sleep fragmentation (e.g., Komada et al., 2006). It is worth noting that in bed-rest studies, isolation and confinement are markedly less pronounced than during space missions, and this makes the attribution of the sleep alterations found in the latter to the isolation and confinement conditions.

The MARS-500 program that more directly explored the effects of isolation and confinement gave mixed results. Indeed, during the first, 105-day isolation study, it has been reported that higher cortisol levels were correlated with the reduction of REM latency, the decrease of sleep duration, and the reduction of delta power in stage 3 sleep (Gemignani et al., 2014). However, during the second, 520-day study, different findings have been reported, with a decreased SWS appearing only after the confinement (Zavalko et al., 2013). In other Mars analog studies, no PSG techniques have been implemented, making it difficult to get informative results.

Data from other settings that share some features with the polar environments and the space missions also contribute to blur the general picture. For instance, studies in underwater settings and Intensive Care Units substantially failed to highlight significant alterations of sleep or of the sleep/wake cycle (e.g., Trousselard et al., 2015; Reinke et al., 2019), whereas studies in caves showed an increased SWS (Mogilever et al., 2018), contrarily to what has been reported in polar settings. Instead, the decrease in SWS has been extensively observed during the exposure to a systemic stressor such as noise (Basner and Samel, 2005; Griefahn et al., 2006), or hypoxia (Rojc et al., 2014; Morrison et al., 2017), even if not conclusive. Although few studies exist, psychosocial stressors have also been found to induce similar changes (Kim and Dimsdale, 2007).

Moreover, several methodological limitations plague the research on this topic. They are probably unavoidable, since the very nature of most of the studies, still they contribute immensely to the unclear pattern of results reported in the literature. Firstly, sample sizes are very small in most of the reviewed studies. This, of course, has a negative impact on the studies’ statistical power, but might also lead to false effects and exacerbation of the impact of how individuals cope with the exceptional situation they live in. Secondly, the effect sizes in most of the studies are also typically small. Again, this might suggest non-specific effects of ICE environments on sleep, or that better experimental control over the multiple variables that might affect the human sleep in ICE environments is mandatory. Thirdly, a huge variety of experimental conditions, protocols, measurements do exist, making it difficult to compare results obtained in different studies and settings.

Most of the constraints that limit the degree of experimental control researchers have over their studies cannot be easily avoided. However, a more systematic and coordinated approach, developing international collaborations (Suedfeld and Weiss, 2000), might be useful. For instance, collecting data in multiple missions/experiments with the same methodology in the same ICE setting would increase the sample size and likely the statistical power, and would reduce the role of all the factors inherent to a specific mission or experiment (e.g., the composition of the crew in terms of individual differences). Also, planning experiments in different and selected ICE conditions to investigate the role of the specific factor under investigation would help to compare the results across different settings. Again, this would help to disentangle the specific role of the several features that define each ICE environment. Future research is required to employ such a more systematic and collaborative approach to solve the several methodological and theoretical limits of the field. Until now, most of the studies on human sleep in ICE environments have only addressed the issue of whether any sleep alteration exists in ICE. It might be time to move toward addressing what causes sleep alterations in ICE environments.

## Conclusion

Overall, the scientific literature on sleep in ICE suggests that sleep alterations in isolated and confined environments are rather non-specific. The general changes in SWS and REM sleep often described due to the prolonged permanence in ICEs are not a specific condition of the prolonged isolation and confinement. These changes appear to be non-specific and generalized responses to various types of stressors. Both systemic and processive stressors that exist in these environments might independently or in a combined way induce changes in sleep architecture and quality, and the current findings do not provide enough information about the direction of these changes. A deeper understanding of the mechanisms underlying such factors is required: until now, circadian and hypoxic effects on sleep represent the most investigated. Nevertheless, the knowledge produced until now seems insufficient to disentangle the spectrum of effects that individuals in chronic stress conditions might experience.

## Author Contributions

All authors listed have made a substantial, direct and intellectual contribution to the work, and approved it for publication. PZ retrieved most of the sources and drafted the initial version of the manuscript.

## Conflict of Interest

The authors declare that the research was conducted in the absence of any commercial or financial relationships that could be construed as a potential conflict of interest.
